# How to Remove a Dumbbell Tumour of the Sciatic Notch

**DOI:** 10.1155/S1357714X00000104

**Published:** 2000-03

**Authors:** Matthew P. Revell, Robert J. Grimer

**Affiliations:** The Royal Orthopaedic Hospital Oncology Service Bristol Road South Birmingham B31 2AP UK

## Abstract

*Purpose.*To look at a method for treating soft tissue sarcoma of the retroperitoneal area.

*Patient.* We report the case of a 38-year-old woman with a well-differentiated liposarcoma.

*Results.* Complete excision was achieved resulting in only minor morbidity and complete local control.


*Sarcoma* (2000) **4**, 61–62

CASE REPORT

### How to remove a dumbbell tumour of the sciatic notch

MATTHEW P. REVELL & ROBERT J. GRIMER

*The Royal Orthopaedic Hospital Oncology Service, Bristol Road South, Birmingham B31 2AP, UK*

**Abstract**
*Purpose*. To look at a method for treating soft tissue sarcoma of the retroperitoneal area.
*Patient*. We report the case of a 38-year-old woman with a well-differentiated liposarcoma.
*Results*. Complete excision was achieved resulting in only minor morbidity and complete local control.

### Introduction

Soft tissue sarcomas of the retroperitoneal area have a fearful prognosis both in terms of local recurrence, mortality and surgical morbidity.[1] Tumours which exit the pelvis into a surrounding area present an even greater challenge to the surgical oncologist. We describe here a novel method for removing a large retroperitoneal sarcoma which had exited through the sciatic notch and presented as a dumbbell tumour.

### Case report

A 38-year-old woman presented with a 12-month history of low back pain and right-sided sciatica. There was a 10 × 10 cm palpable mass in the right buttock and there were signs of sciatic nerve irritation with weakness of extensor hallucis longus, altered sensation on the outer side of the foot and an absent ankle jerk. Imaging with MRI revealed an 18 × 7 × 9 cm tumour extending from within the pelvis, through the sciatic notch into the buttock (Fig. 1). Biopsy confirmed this to be a well-differentiated liposarcoma.

At the time of definitive surgery the tumour was removed by an anterior approach osteomizing the pelvis from the sciatic notch anteriorly. The greater trochanter was also osteomized and the hip abductors elevated superiorly on their neurovascular bundle. By distracting the two parts of the pelvic osteotomy the whole of the tumour could now be visualized with the sciatic nerve stretched across it. The tumour could now be removed from the surrounding structures without tumour spill. However, a small area of the pseudocapsule was removed separately as it involved the sciatic nerve. The ilium was internally fixed with two plates and the trochanter rewired as in a hip replacement (Fig 2).

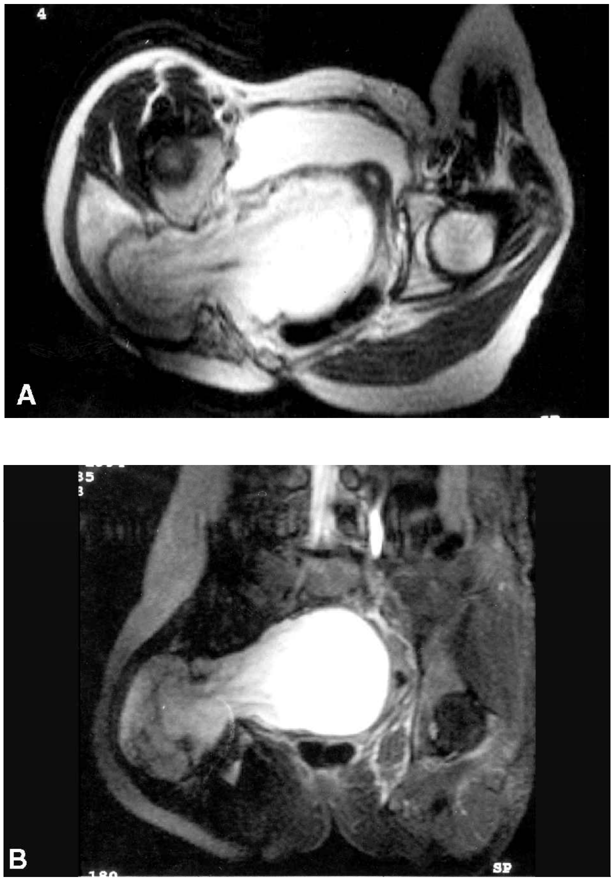

**Figure 1**. *MRI scans to show the tumour traversing the sciatic notch in transverse (a) and coronal (b) section.*

The postoperative recovery was marred by a wound infection with MRSA which responded to antibiotics. After 6 weeks of protected weight-bearing, she

Correspondence to: Robert J. Grimer, The Royal Orthopaedic Hospital Oncology Service, The Royal Orthopaedic Hospital, Bristol Road South, Northfield, Birmingham B31 2AP, UK. Tel: (+44) 121-685-4150; Fax: (+44) 121-685-4146; E-mail: sarcomas@rohos.demon.co.uk

1357-714X print/1369-1643 online/00/010061-02 © 2000 Taylor & Francis Ltd

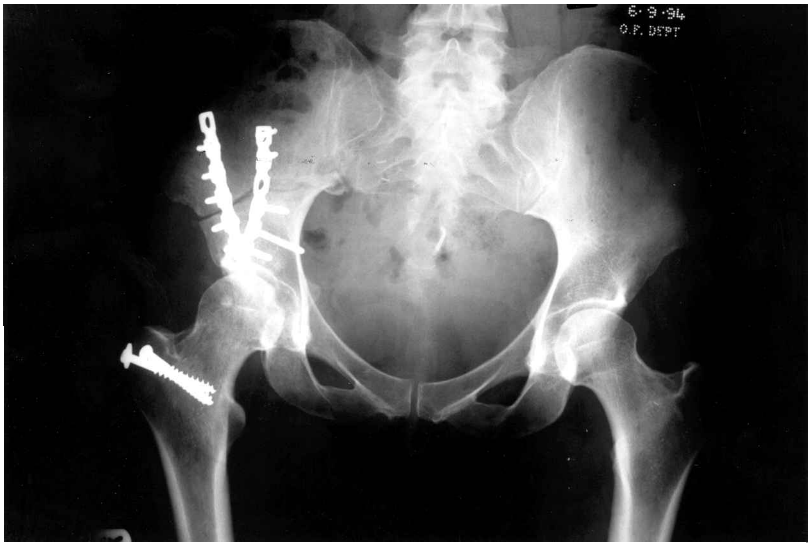

**Figure 2.** *The immediate postoperative X-ray demonstrating the two osteotomies.*

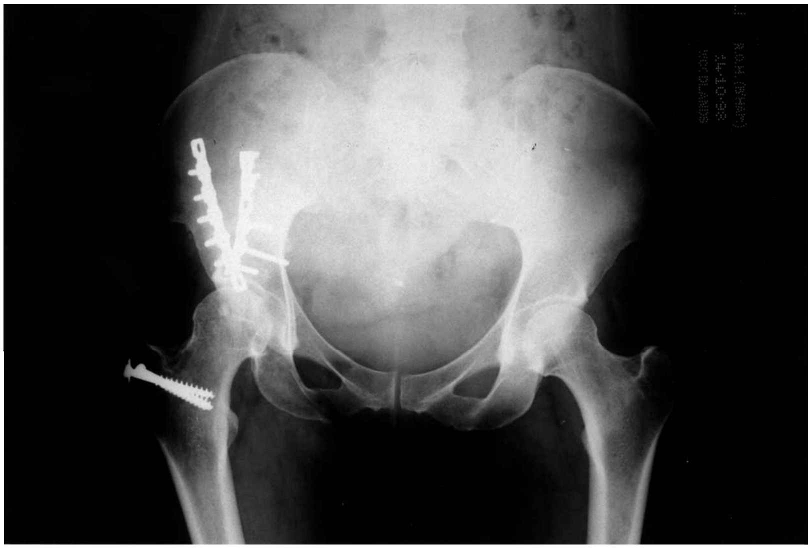

**Figure 3.** *An X-ray taken at 5 years demonstrating complete union of the pelvic and trochanteric osteotomies.*

progressed to walking sticks. Histological examination confirmed the diagnosis of myxoid liposarcoma. Margins were clear other than the small intralesional area near the sciatic nerve. It was decided not to treat her with adjuvant chemotherapy or radiotherapy.

After 5 years she remains disease-free but still has some weakness of the right leg with reduced power on hip flexion, requiring one walking stick. She also suffers from low back pain from a degenerate spine.

### Discussion

This novel approach to a dumbbell tumour of the sciatic notch has achieved the aim of surgical oncology for this soft tissue sarcoma—complete excision with minimal morbidity and complete local control. The option of splitting bones to access tumours is not new to thoracic surgeons but is not usually considered for tumours of the pelvis. This approach may be considered for unusually large dumbbell tumours. It is possible to remove a smaller lesion through a combination of a retroperitoneal anterior ilio-inguinal approach with a second posterior gluteus maximus splitting approach. We hope that this case report will stimulate others to consider such methods when tackling tumours in this situation in the future.

### Reference

1 Watkins RM, Thomas JM. Role of computed tomography in selecting patients for hindquarter amputation. *British Journal of Surgery* 1987;78(8):711–4.
